# ENDOSCOPIC EVALUATION OF POST-FUNDOPLICATION ANATOMY AND CORRELATION WITH SYMPTOMATOLOGY

**DOI:** 10.1590/0102-672020200003e1543

**Published:** 2021-01-15

**Authors:** Bruno Costa MARTINS, Clarissa Santos SOUZA, Jennifer Nakamura RUAS, Carlos Kiyoshi FURUYA, Sonia Nadia FYLYK, Christiano Makoto SAKAI, Edson IDE

**Affiliations:** 1 Endoscopy Unit, Oswaldo Cruz German Hospital, São Paulo, SP, Brazil

**Keywords:** Fundoplication, Endoscopy, Gastroesophageal reflux disease, Digestive system surgical procedures, Fundoplicatura, Endoscopia digestiva, Doença do refluxo gastroesofágico, Cirurgia do aparelho digestivo

## Abstract

**Background:**

Upper digestive endoscopy is important for the evaluation of patients submitted to fundoplication, especially to elucidate postoperative symptoms. However, endoscopic assessment of fundoplication anatomy and its complications is poorly standardized among endoscopists, which leads to inadequate agreement.

**Aim:**

To assess the frequency of postoperative abnormalities of fundoplication anatomy using a modified endoscopic classification and to correlate endoscopic findings with clinical symptoms.

**Method:**

This is a prospective observational study, conducted at a single center. Patients were submitted to a questionnaire for data collection. Endoscopic assessment of fundoplication was performed according to the classification in study, which considered four anatomical parameters including the gastroesophageal junction position in frontal view (above or at the level of the pressure zone); valve position at retroflex view (intra-abdominal or migrated); valve conformation (total, partial, disrupted or twisted) and paraesophageal hernia (present or absent).

**Results:**

One hundred patients submitted to fundoplication were evaluated, 51% male (mean age: 55.6 years). Forty-three percent reported postoperative symptoms. Endoscopic abnormalities of fundoplication anatomy were reported in 46% of patients. Gastroesophageal junction above the pressure zone (slipped fundoplication), and migrated fundoplication, were significantly correlated with the occurrence of postoperative symptoms. There was no correlation between symptoms and conformation of the fundoplication (total, partial or twisted).

**Conclusion:**

This modified endoscopic classification proposal of fundoplication anatomy is reproducible and seems to correlate with symptomatology. The most frequent abnormalities observed were slipped and migrated fundoplication, and both correlated with the presence of symptoms.

## Graphical Abstract

[Fig f1a]

**Figure f1a:**
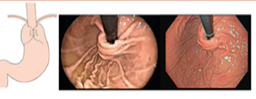
Endoscopic aspect of fundoplication anatomy: Normal aspect of Nissen fundoplication

## RESUMO

**Racional:**

A endoscopia digestiva alta é importante ferramenta para a avaliação de pacientes submetidos à fundoplicatura, principalmente para elucidar os sintomas pós-operatórios. Entretanto, a avaliação endoscópica da sua anatomia e complicações é atualmente pouco padronizada entre os endoscopistas, o que leva à disparidade de laudos e condutas.

**Objetivo:**

Avaliar a frequência de anormalidades pós-operatórias da fundoplicatura através de uma classificação endoscópica e correlacionar os achados endoscópicos com os sintomas clínicos.

**Método:**

Este é estudo observacional prospectivo, realizado em um único centro. Os pacientes foram submetidos a um questionário para coleta de dados. A avaliação endoscópica da fundoplicatura foi realizada de acordo com a classificação em estudo, que considerou quatro parâmetros anatômicos, incluindo a posição da junção gastroesofágica em vista frontal (acima ou no nível da zona de pressão); posição da válvula na visão retroflexa (intra-abdominal ou migrada); conformação valvar (total, parcial, desgarrada ou torcida) e hérnia paraesofágica (presente ou ausente).

**Resultados:**

Foram avaliados 100 pacientes submetidos à fundoplicatura, 51% homens (idade média: 55,6 anos). Quarenta e três por cento relataram sintomas pós-operatórios. Anormalidades endoscópicas da anatomia da fundoplicatura foram relatadas em 46% dos pacientes. Junção gastroesofágica acima da zona de pressão (fundoplicatura deslizada) e fundoplicatura migrada foram significativamente correlacionadas com a ocorrência de sintomas pós-operatórios. Não houve correlação entre sintomas e conformação da fundoplicatura (total, parcial ou torcida).

**Conclusão:**

Essa classificação endoscópica modificada proposta para avaliar a anatomia da fundoplicatura é reprodutível e parece correlacionar-se com a sintomatologia. As anormalidades mais frequentes observadas foram fundoplicaturas migradas e deslizadas, e ambas se correlacionaram com a presença de sintomas.

## Graphical Abstract

[Fig f1as]

**Figure f1as:**
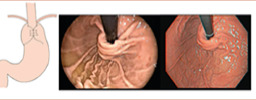
Aspecto endoscópico da anatomia da fundoplicatura: aspecto normal da fundoplicatura de Nissen

## INTRODUCTION

Gastroesophageal reflux disease (GERD) is the most common upper gastrointestinal benign disease in Western countries, with a prevalence of 10-20%^6^ ^,^ ^20^ ^,^ ^22^ ^,^ ^26^. The role of surgery in the treatment is well established. Laparoscopic fundoplication (LFP) is considered an effective alternative to drug treatment and is associated with excellent short and long-term outcomes (80-95%)^3^ ^,^ ^4^ ^,^ ^5^ ^,^ ^7^ ^,^ ^17^ ^,^ ^23^ ^,^ ^24^ ^,^ ^28^. The goal of surgical treatment is to make a symmetrical fundoplication around the distal esophagus, situated below the closed hiatus (hiatoplasty)^21^.

Concurrent with the increase number of LFP over the last two decades^8,14,16,21^ the number of dissatisfied patients, presenting relapses or new symptoms after surgery has also arrised^2^ ^,^ ^11^ ^,^ ^16^ ^,^ ^21^. Given this scenario, precise endoscopic evaluation of valve fundoplication is extremely important because it allows the medical team to recognize abnormalities and to establish treatment strategies, especially if surgical reintervention is being considered^21^ ^,^ ^25^. However, there is currently no definitive standardization in the description of endoscopic findings and most of the analyzes are subjective, with poor agreement among endoscopists^10^ ^,^ ^16^ ^,^ ^21^. Therefore, endoscopic reports adds few information to guide surgeons about abnormalities present.

Mittal et al^21^ proposed a classification of endoscopic findings of fundoplications based on the evaluation of four anatomical parameters that could identify postoperative abnormalities: the gastroesophageal junction location and its relation to the crura, relationship of the fundoplication to the gastroesophageal junction (GEJ), a description of the fundoplication, and any sign of paraesophageal hernia. The authors judge that this classification was applicable to previous endoscopic reports, with good correlation with symptoms^21^. 

Considering the need for greater uniformity of endoscopic description of fundoplications, we implemented an endoscopic classification in our service, based on the classification proposed by Mittal et al^21^, with some modifications in order to facilitate its applicability. 

The objective of this study was to access the frequency of post-fundoplication abnormalities using a simplified and objective endoscopic criteria evaluation and to analyze if there is any correlation between endoscopic abnormalities and symptomatology.

## METHOD

This study was approved by the ethical committee of our hospital and all included patients freely signed an informed consent form.

From September 2014 to July 2015, all patients scheduled to routine upper endoscopy who had been submitted to fundoplication surgery (open or laparoscopic) were included in this study. At our endoscopy division, we receive patients from all over the country, so fundoplication surgery could have been performed anywhere else. Before endoscopy, they were submitted to a questionnaire to collect data regarding gender, age, pre and postoperative symptoms, postoperative interval after fundoplication and whether or not a reoperation had occurred. After the interview, patients were submitted to conventional upper gastrointestinal endoscopy, with an objective evaluation of the fundoplication. 

Upper endoscopies were performed by all doctors of our endoscopy team (five endoscopists), which were trained to apply the referred classification. After obtaining personal data and individualized classification of fundoplications, statistical analyzes were performed to evaluate the occurrence of LFP abnormalities and to correlate clinical and endoscopic findings.

### Endoscopic evaluation of fundoplication (Figure 1)

#### GEJ position in frontal view

##### GEJ located at the pressure zone

Pressure zone is composed by crura plus fundoplication valve (endoscopically separation of these two structures is usually not possible). This means that the GEJ is surrounded by the fundoplication valve (admitted as normal if extending ≤1 cm above the pressure zone), as shown in Figure 2A.

##### GEJ above the pressure zone

Located 1-2 cm above the pressure zone.

##### Slipped fundoplication

GEJ >2 cm above the pressure zone, simulating a hiatal hernia. This situation represents a slipped fundoplication, which instead of involving the Z line, involves the stomach itself (gastrogastric fundoplication), as seen in Figure 2F.

#### Fundoplication position/situation (at retroflex view)

##### Intra-abdominal fundoplication

Valve fundoplication located below hiatoplasty.

##### Partially migrated fundoplication

Valve fundoplication partially migrated towards the thorax, due to a loose or partially disrupted hiatoplasty.

##### Fully migrated fundoplication

Valve fundoplication fully migrated to the thorax, above the diaphragmatic, as depicted in Figure 2E.

#### Conformation of fundoplication (at retroflex view)

##### Total (or complete) fundoplication

The transverse gastric folds circumferentially involve the cardia as observed in retroflexed position, remaining juxtaposed to the endoscope shaft in all phases of respiration. The fundoplication creates a nipple valve with stacked coil appearance about 3 cm long that should rest parallel to the white distance lines of the endoscope^20^. This conformation resembles the letter U (Figure 2A). Very thick fundoplications may cause dysphagia while very thin ones may not be effective.

##### Partial fundoplication

The transverse gastric folds partially involve the cardia. Its conformation resembles the Greek letter “omega” and exposure of the Z-line may occur with breathing movements^20^. This finding may represent a partial disruption of the Nissen LFP valve (originally complete - 360°) or correspond to the Toupet-Lind LFP in which a valve that partially involves the cardia (270°) is intentionally made. Thus, this conformation does not always mean an “abnormality” (Figure 2B).

##### Disrupted fundoplication

The transverse gastric fold does not involve the endoscope shaft. Sometimes the gastric fold is not even perceptible. Occasionally it can be seen making a straight line at the cardia (Figure 2C).

##### Twisted fundoplication

The gastric fold is in an oblique position and is not parallel to the endoscope’s demarcation lines. A craniocaudal axis deviation of the fundoplication valve is seen (Figure 2D).

#### Paraesophageal hernia

##### No paraesophageal hernia is the expected situation

###### Presence of paraesophageal hernia

Although valve fundoplication is intact, the hiatoplasty may be enlarged, allowing fundus tissue to slide towards the thorax (Figure 2G).

**FIGURE 1:**
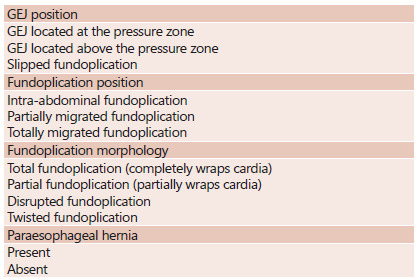
Endoscopic evaluation of fundoplication anatomy

GEJ=gastroesophageal junction

**FIGURE 2:**
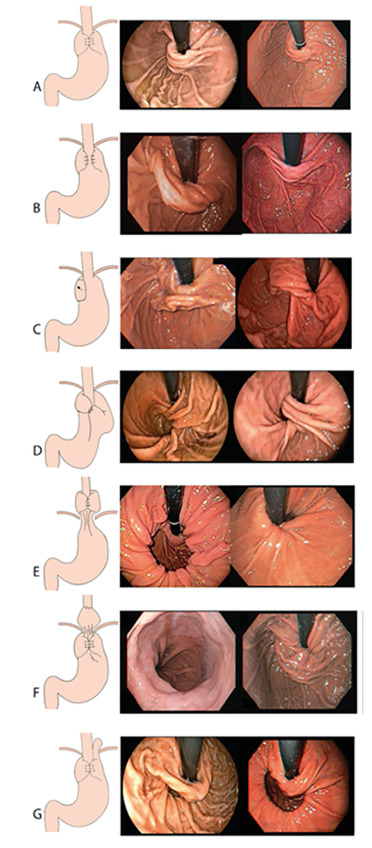
Endoscopic aspect of fundoplication anatomy: A) normal aspect of Nissen fundoplication; B) partial fundoplication; C) disrupted fundoplication; D) twisted fundoplication; E) migrated fundoplication; F) slipped fundoplication; G) paraesophageal hernia.

In summary, the “ideal” fundoplication (Figure 2A) should present: 1) GEJ near or at the pressure zone; 2) intra-abdominal position; 3) partially or totally involving the cardia; and 4) no paraesophageal hernia.

Original surgical reports were not available, as patients could have been operated outside our hospital. (For the sake of better understanding our results, the reader must be aware that the most common technique performed in Brazil is Nissen, followed by Toupet-Lind^19^. Anterior fundoplication is extremely rare in our country).

### Statistical analysis

Categorical variables were presented in figures and in tables containing absolute (n) and relative (%) frequencies. The association between these variables was assessed using the Chi-squared test or Fisher’s exact test. Calculations were performed by StatPlus: mac software (AnalystSoft Inc.). 

## RESULTS

Of the 100 evaluated patients, 51 were male; mean age was 55.6 years (15-87y). Most patients (56) had fundoplication performed less than five years before endoscopy; 20 were operated 6 to 10 years before, and 24 had surgery more than 10 years earlier. Only eight patients were submitted to reoperation.

The most frequent preoperative symptoms were heartburn (73%) and regurgitation (59%). At the time of endoscopy, the majority (57%) had no symptoms, as shown in Table 1.

**TABLE 1:** Prevalence of preoperative and postoperative symptoms.

Symptoms (n=100)	Preoperative symptoms (%)	Postoperative symptoms (%)
Heartburn	73	23
Regurgitation	59	8
Retrosternal pain	32	5
Cough	12	3
Abdominal pain	4	6
Dysphagia	3	12
Asymptomatic	2	57

### Characteristics of fundoplication

With respect to endoscopic evaluation, fundoplication was adequate in more than 70% of the cases for the four parameters used: 1) position of the GEJ; 2) fundoplication position; 3) fundoplication conformation; and 4) presence of paraesophageal hernia.

The most common abnormalities were: GEJ above pressure zone or slipped (28%), fundoplication migration (25%) and partial fundoplication (16%). Only 4% had paraesophageal hernia. These results can be seen in Table 2.

**TABLE 2:** Evaluation of the fundoplication parameters

Evaluated parameters	Observed frequency
GEJ position	
At the pressure zone ^†^	72%
Above pressure zone or slipped	28%
Fundoplication position	
Intra-abdominal^†^	75%
Partially migrated	12%
Totally migrated	13%
Fundoplication conformation	
Total^†^	75%
Partial	16%
Disrupted	7%
Twisted	2%
Paraesophageal hernia	
Absent^†^	96%
Present	4%

^† =^parameters indicating “ideal” fundoplication

### Correlation between symptomatology and fundoplication anatomy 

**FIGURE 3:**
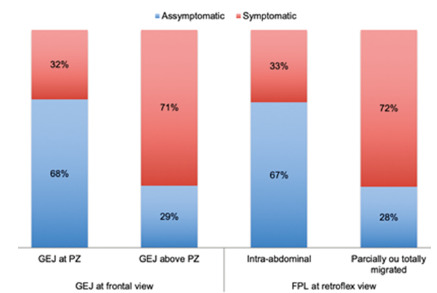
Correlation between the presence of symptoms and the endoscopic evaluation: position of the GEJ at frontal view and position of the fundoplication at retroflex view.

The number of asymptomatic patients when the GEJ was at the pressure zone was significantly higher than when the GEJ was above it or slipped (68% vs. 28.5%, p<0.001), as shown in Figure 3. The same was observed when we assessed fundoplication position. The number of asymptomatic patients was higher when fundoplication was intra-abdominally, compared to partially or totally migrated fundoplications (66.6% vs. 28.0%, p<0.001).

In contrast, there was no statistical significance between fundoplication conformations (total, partial, disrupted or twisted) and symptomatology. Of the patients with total LFP, 60.8% reported being asymptomatic vs. 46.1% with other descriptions (p=0.19).

PZ=pressure zone; GEJ=gastroesophageal junction; FPL=fundoplication

## DISCUSSION

Studies analyzing long-term results of anti-reflux surgery, with follow-ups varying from 5-12 years, reveal that 2-30% of patients may present recurrence, persistence or new symptoms related to gastroesophageal reflux disease^12^. In this scenario, a precise endoscopic evaluation is critical for diagnostic elucidation. In our study, 43% of patients reported one or more postoperative symptoms, and we found an association between symptomatology and fundoplication abnormalities.

Upper digestive endoscopy is generally indicated as the first diagnostic test to evaluate fundoplication^15^. However, endoscopists have difficulty evaluating FPL anatomy, specially FPL abnormalities, which may be overlooked or underestimated. This might be in part by fear of criticizing the surgeon, but also because of a lack of adequate standardization for evaluation. The concept that FPL abnormalities are better diagnosed by contrast studies than by endoscopy is incorrect. As a matter of fact, radiologists may even have more difficulty than endoscopists to fully understand the fundoplication anatomy. In fact, a study evaluating fundoplication integrity in 22 patients with postoperative symptoms showed that the abnormalities described by endoscopy were twice higher than the abnormalities described by esophagogram^13^.

Familiarity with fundoplication surgical steps seems to be important for its correct evaluation. Juhasz et al,^16^ compared the endoscopic findings of fundoplication abnormalities reported by outside endoscopists (community gastroenterologists) vs. the authors’ findings (esophageal center). Only 32% of outside physicians reported the presence of previous fundoplication. Furthermore, outside physicians diagnosed fewer slipped fundoplications (9/208 vs. 52/208, p<0.001) and fewer twisted fundoplications (18/208 vs. 61/208, p<0.001), compared to experienced endoscopists.

Jobe et al^15^ uphold that the best method to evaluate a fundoplication is through endoscopy. They made the first attempt to describe the endoscopic findings of normal successful fundoplications according to the different techniques and proposed an endoscopic lexicon to report these criteria. In their study, endoscopic images of fundoplications of asymptomatic patients were analyzed. From the evaluation of these images by experienced surgeons and gastroenterologists, ten criteria were elaborated to define an ideal fundoplication. They concluded that these criteria could be used during the evaluation of patients with complaints of the upper gastrointestinal tract after anti-reflux surgery and serve as a reliable evaluation method.

Mittal et al^21^ evaluated symptomatic post-fundoplication patients who underwent reoperative antireflux surgeries and proposed a classification system to standardized endoscopic reports of fundoplication anatomy, demonstrating a good correlation to symptomatology. This study found out that chest pain and dysphagia were more associated to twisted fundoplication due to distal obstruction, while regurgitation and recurrent heartburn were frequently seen in disrupted fundoplication. In addition, the presence of slipped fundoplication tended to represent a complete failure of treatment and could be associated with any symptomatology evaluated. This system was proposed mainly to categorize patients who had failed fundoplication^1^ ^,^ ^21^.

The classification proposed by Mittal et al^21^ was very helpful to understand post-fundoplication abnormalities; however, some aspects remained doubtful and could limit its applicability by a large number of endoscopists. The terms “partially disrupted fundoplication” should be avoided unless a previous endoscopy report have documented with images a well assembled total valve; “partial valve” (or “partial fundoplication)” is more adequate in this situation, since if we´re examining a patient submitted to Lind-Toupet technique, this would be the normal aspect expected. Mittal et al^21^ argue that is important to differentiate the distance between GEJ and fundoplication from the distance between GEF and crura. Nevertheless, it is very difficult to make this distinction in practice. This difficulty can be overcome by analyzing a slipped fundoplication in the frontal view and a migrated valve at retroflex view. The subclassification F2b, described as two-compartment stomach, has no further explanation or illustrations to explain it, what could generate misunderstanding. This subclassification was suppressed in our system. Moreover, we gave preference to nominate the anatomical abnormalities instead of codifying it with letters and numbers, since we considered it easier to memorize this way. 

In our study, we also observed that endoscopic evaluation of fundoplications helps to correlate the presence of symptoms and anatomical findings. Of the 100 fundoplication patients evaluated, 46% had some endoscopic surgery abnormality. When correlating the presence of symptoms with fundoplication evaluation, we observed that the GEJ outside the pressure zone and migrated fundoplication were significantly associated with the occurrence of symptoms (p<0.001 in both cases).

Koch et al^18^ showed that Nissen and Toupet fundoplication procedures resulted in an expressive increase in lower esophageal sphincter pressure, but the improvement was significantly greater with the Nissen procedure^18^. However, postoperative results were similar in both surgeries, indicating that not only the lower esophageal sphincter pressure is important but also the adequate restoration of the gastroesophageal valve^9^. In our study, there was no correlation between the presence of symptoms and the conformation of the fundoplication (whether total, partial or twisted, p=0.19).

This study has important limitations, especially the small number of patients included in the analysis and the fact that it was carried out in a single center. Unfortunately, we didn’t had access to original surgical reports nor previous control endoscopies. However, this may have had minimal impact in our results as our main goal was to describe the current endoscopic anatomy and knowing the original surgical technique could have biased our description. Moreover, partial or total fundoplication had no correlation with symptoms. On the other hand, previous endoscopies reports could have helped in the diagnostic of valve disruption or migration. Additional information, such as correlation to manometry and pH study of the symptomatic group would bring important information in order to discriminate the symptoms directly related to GERD from functional gastrointestinal disorders not related to anti-reflux surgery. Another aspect not analyzed in this study was the fundoplication thickness. Ideal Nissen fundoplication should have a nipple valve about 2-3 cm long. Thinner fundoplication could be related to persistence or early relapse of reflux symptoms, and thicker fundoplication may relate to dysphagia.

## CONCLUSION

This standardized endoscopic classification of fundoplication anatomy is reproducible and seems to correlate with symptomatology. The most frequent abnormalities observed were slipped and migrated fundoplication, and both correlated with the presence of postoperative symptoms. There was no correlation between symptoms and endoscopic findings of total or partial fundoplication.
